# Supplementary material for: Theoretical Study of Zirconium Isomorphous Substitution into Zeolite Frameworks
Source: Molecules. 2019 Dec 5;24(24):4466. doi: 10.3390/molecules24244466 (PMC6943428; doi:10.3390/molecules24244466)
Supplement: Supplementary file 1 [file molecules-24-04466-s001.pdf]

Supplementary File

Theoretical Study of Zirconium Isomorphous Substitution into Zeolite Frameworks

Duichun Li [1], Bin Xing [1], Baojun Wang [2,*] and Ruifeng Li [1,*]

[1] College of Chemistry and Chemical Engineering, Taiyuan University of Technology, Taiyuan 030024, China; liduichun1985@163.com (D.L.); xingbin@tyut.edu.cn (B.X.)

[2] Key Laboratory of Coal Science and Technology of Ministry of Education and Shanxi Province, Taiyuan University of Technology, Taiyuan 030024, China

* Correspondence: wangbaojun@tyut.edu.cn (B.W.); rfli@tyut.edu.cn (R.L.)

Contents:

Equations 2

The substitution energies (E_{sub}) 2

The deprotonation energies (E_{DPE}) 2

The electrostatic potential (ν) 2

The structural distortion (Θ and Ω) 3

Figures 4

Figure S1. The relative energies (ΔE/eV) of different T site for Zr atom substitution in the SOD framework with Si/Al ratio from 11 to 1. 4

Figure S2. The most stable configurations of a) one, b) two, c) three, d) four, e) five and f) six Zr atoms substitution on the basis of SOD framework with Si/Al=11, Zirconium (Blue-green).5

Figure S3. The most stable configurations of a) one, b) two, c) three, d) four, e) five and f) six Zr atoms substitution on the basis of SOD framework with Si/Al = 5 ~ 1. 7

Figure S4. The most stable configurations of AlPO-34-$Zr_{1\sim6}$. 8

Figure S5. The most stable configurations of SAPO-34-$Zr_{1\sim6}$. 9

1. Equations

1.1. Substitution Energies (E_{sub})

Because the distribution of substituted T sites in frameworks is determined by the thermodynamic stability of molecular sieve frameworks [1,2], the substitution energy (E_{sub}) is an appropriate criterion for evaluating the energetic priority for a specific T site substitution by a heteroatom. The following virtual reaction is carried out to represent the substitution process:

$$(O\text{-}Si\text{-}O)_n^{SOD} + nAl(OH)_3 + nH_2O \rightarrow (O\text{-}Al\text{-}OH)_n^{SOD} + nSi(OH)_4 \quad (1)$$

$$(O\text{-}Si\text{-}O)_n^{SOD} + nZr(OH)_4 \rightarrow (O\text{-}Zr\text{-}O)_n^{SOD} + nSi(OH)_4 \quad (2)$$

$$(O\text{-}P\text{-}O)_n^{AlPO/SAPO} + nZr(OH)_4 \rightarrow (O\text{-}Zr\text{-}OH)_n^{AlPO/SAPO} + nH_3PO_4 \quad (3)$$

Also, one can define the substitution energies (E_{sub}) as follows [3,4]:

$$E_{sub\text{-}nAl} = E_{(O\text{-}Al\text{-}OH)_n^{SOD}} + nE_{Si(OH)_4} - E_{(O\text{-}Si\text{-}O)_n^{SOD}} - nE_{Al(OH)_3} - nE_{H_2O} \quad (4)$$

$$E_{sub\text{-}nZr} = E_{(O\text{-}Zr\text{-}O)_n^{SOD}} + nE_{Si(OH)_4} - E_{(O\text{-}Si\text{-}O)_n^{SOD}} - nE_{Zr(OH)_4} \quad (5)$$

$$E_{sub\text{-}nZr} = E_{(O\text{-}Zr\text{-}OH)_n^{AlPO/SAPO}} + nE_{H_3PO_4} - E_{(O\text{-}P\text{-}O)_n^{AlPO/SAPO}} - nE_{Zr(OH)_4} \quad (6)$$

where $E_{sub\text{-}nAl/Zr}$ corresponds to the substitution energies of the optimized SOD, AlPO-34, and SAPO-34 framework substituted by Al or Zr atoms; $E_{(O\text{-}Si\text{-}O)_n^{SOD}}$ and $E_{(O\text{-}P\text{-}O)_n^{AlPO/SAPO}}$ are the energies of the SOD, AlPO-34, SAPO-34 frameworks, respectively; $E_{(O\text{-}Zr\text{-}O)_n^{SOD}}$ and $E_{(O\text{-}Zr\text{-}OH)_n^{AlPO/SAPO}}$ are the energies of the corresponding Zr-substituted SOD, AlPO-34, and SAPO-34 frameworks, respectively. $E_{Si(OH)_4/Zr(OH)_4/H_3PO_4}$ is the energy of the virtual complexes.

The average substitution energy ($E_{av\text{-}sub}$) is used in this work to compare the difficulty of substitutions with different Zr content in the framework

$$E_{av-sub} = E_{sub}/n \quad (7)$$

1.2. Deprotonation Energies (E_{DPE})

The deprotonation energy (E_{DPE}) is defined as follows [5]:

$$(O\text{-}Al\text{-}OH)^{SOD}/(O\text{-}Zr/Si\text{-}OH)^{AlPO/SAPO} \rightarrow (O\text{-}Al\text{-}O^-)^{SOD}/(O\text{-}Zr/Si\text{-}O^-)^{AlPO/SAPO} + H^+$$

$$E_{DPE} = E_{(O\text{-}Al\text{-}O^-)^{SOD}/(O\text{-}Zr/Si\text{-}O^-)^{AlPO/SAPO}} + E_{H^+} - E_{(O\text{-}Al\text{-}OH)^{SOD}/(O\text{-}Zr/Si\text{-}OH)^{AlPO/SAPO}} \quad (8)$$

where $E_{(O\text{-}Al\text{-}O^-)^{SOD}/(O\text{-}Zr/Si\text{-}O^-)^{AlPO/SAPO}}$, E_{H^+} , and $E_{(O\text{-}Al\text{-}OH)^{SOD}/(O\text{-}Zr/Si\text{-}OH)^{AlPO/SAPO}}$ are the respective energies of the framework anion after deprotonation, the gaseous proton, and the neutral protonated form of the molecular sieve.

1.3. Electrostatic Potential (ν)

ν is calculated as:

$$\nu = \frac{1}{4\pi\varepsilon}\sum_{i=1}^{4}\frac{q \times q_i}{r_i} = A\sum_{i=1}^{4}\frac{q \times q_i}{r_i} \quad (9)$$

where ε is the electrostatic constant, q and qi are the charges (|e|) of the Al atom and neighboring O atoms, and ri is the bond length (Å) of the four Al-O bonds. In this work, we mainly consider the relative value of ν, and thus the $\frac{1}{4\pi\varepsilon}$, q and r_i are contributed to A.

1.4. Structural Distortion (Θ and Ω)

The local structural perturbations of $[TO_4]$ (T = Al, Zr) from a regular tetrahedron using the root mean square deviation parameter (Θ), which is defined as [6,7]:

$$\Theta = \sqrt{\frac{1}{6}\sum_{i=1}^{6}\left(\alpha_i - \bar{\alpha}\right)^2} \quad (10)$$

where α_i represents the ith Θ(O-T-O) angle and $\bar{\alpha}$ is the average of the six Θ(O-T-O) angles. The Θ is not a direct measure of the distortion caused by the incorporation of heteroatoms into the framework positions of zeolites [8]. The changes in local $[TO_4]$ geometries caused by the heteroatom substitution can be quantified using the parameter Ω, which is defined as [8]:

$$\Omega_{Si \to Al} = \left(\Theta_{Al} - \Theta_{Si}\right)/\Theta_{Si} \quad (11)$$

$$\Omega_{Si \to Zr} = \left(\Theta_{Zr} - \Theta_{Si}\right)/\Theta_{Si} \quad (12)$$

$$\Omega_{P \to Zr} = \left(\Theta_{Zr} - \Theta_{P}\right)/\Theta_{P} \quad (13)$$

where Θ_{Al}, Θ_{Zr}, Θ_{Si}, and Θ_{P} are the root mean square deviations from regular tetrahedron for the Al/Zr atom substitution in SOD, AlPO-34, and SAPO-34 frameworks.

2. Figures

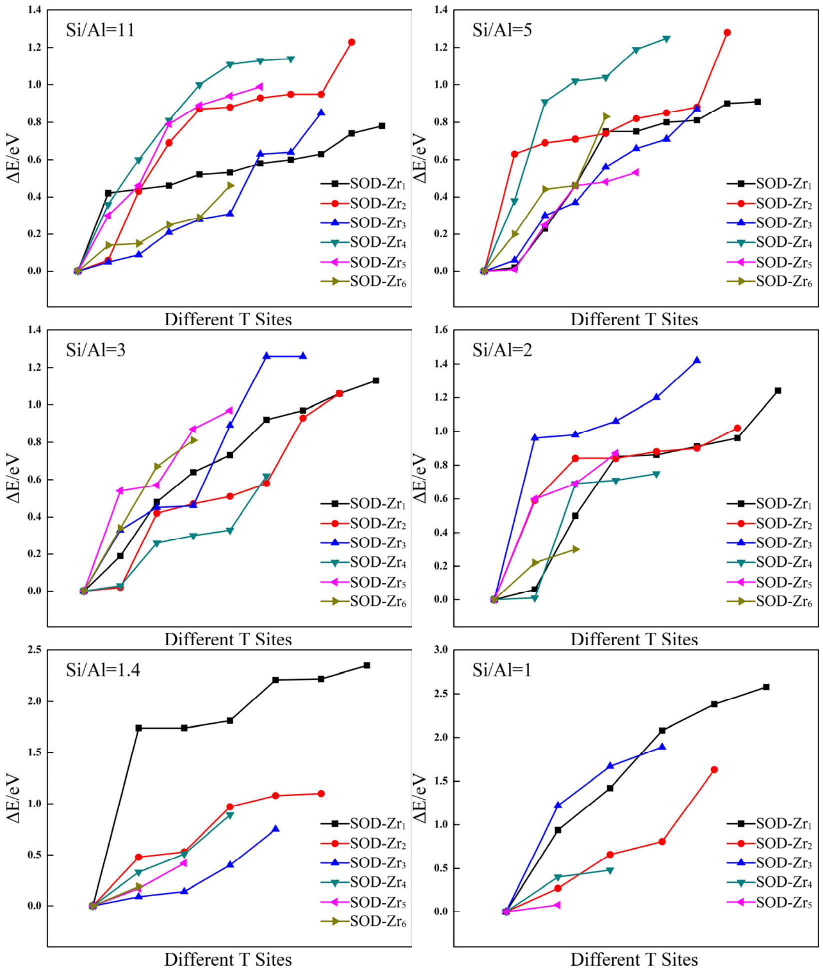

Figure S1. The relative energies (ΔE/eV) of different T site for Zr atom substitution in the SOD framework with Si/Al ratio from 11 to 1.

The location sites of Zr atom in the framework can refer to Figure 1 in the manuscript which shows location of Tn sites in the framework.

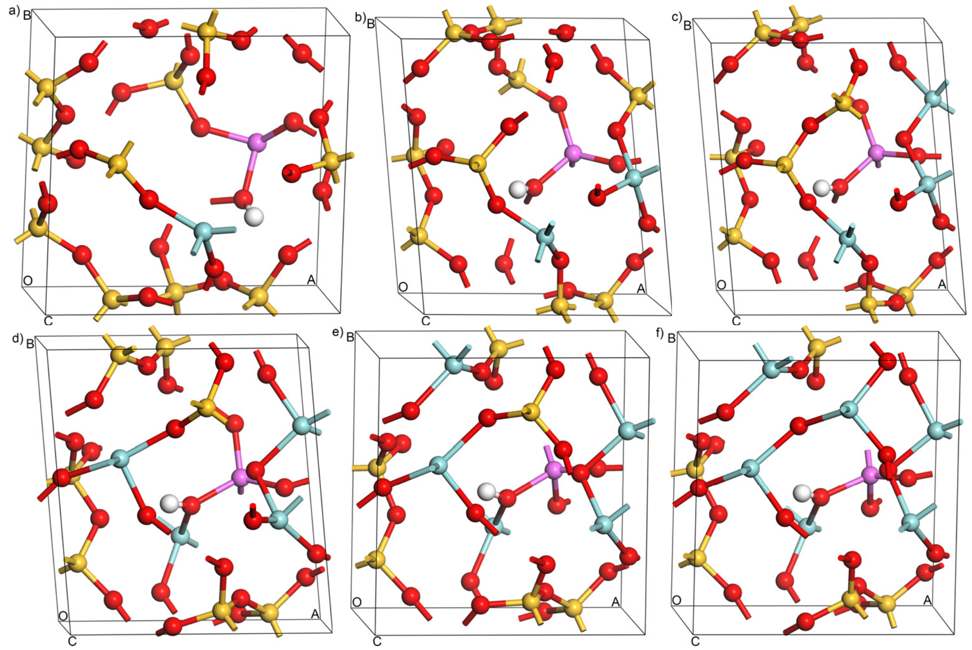

Figure S2. The most stable configurations of (**a**) one, (**b**) two, (**c**) three, (**d**) four, (**e**) five, and (**f**) six Zr atoms substitution on the basis of SOD framework with Si/Al = 11. Zirconium is blue-green.

The location sites of Zr atom in the framework can refer to Figure 1 in the manuscript which shows the location of Tn sites in the framework.

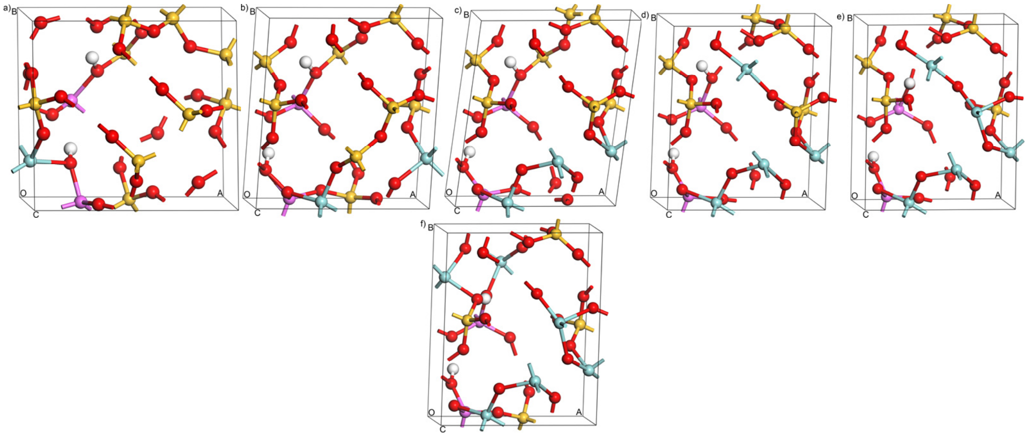

Si/Al = 5

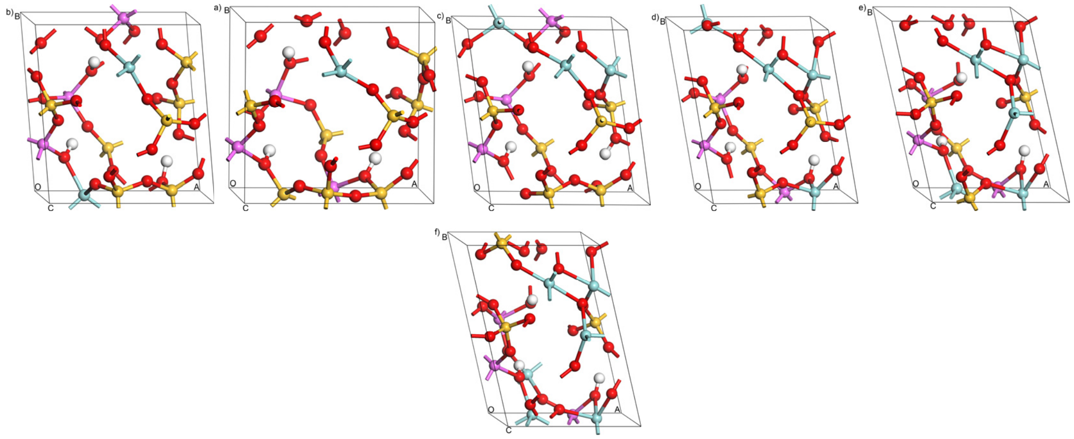

Si/Al = 3

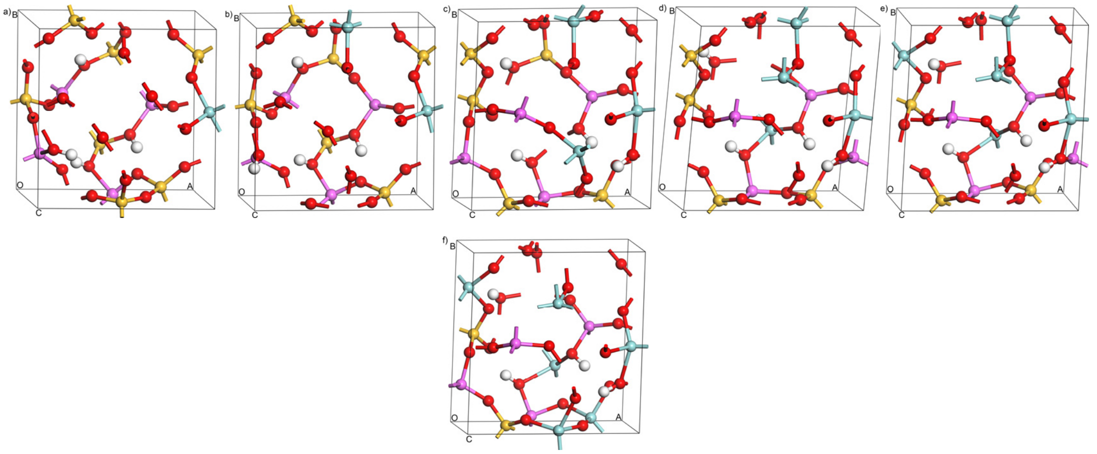

Si/Al = 2

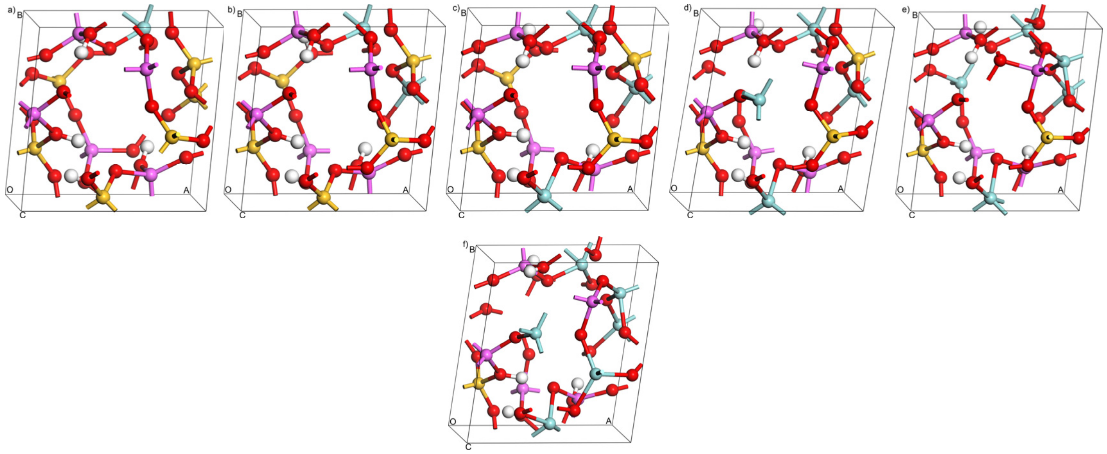

Si/Al = 1.4

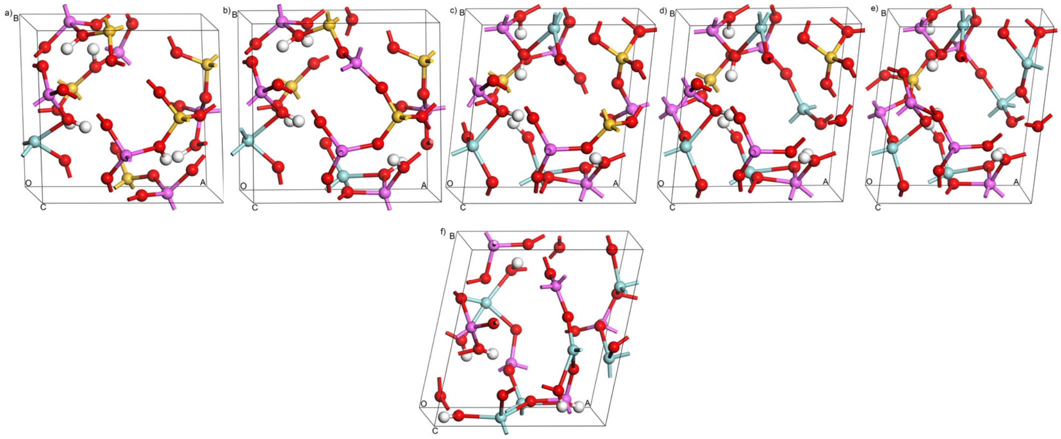

Si/Al = 1

Figure S3. The most stable configurations of (**a**) one, (**b**) two, (**c**) three, (**d**) four, (**e**) five, and (**f**) six Zr atoms substitution on the basis of SOD framework with Si/Al = 5~1.

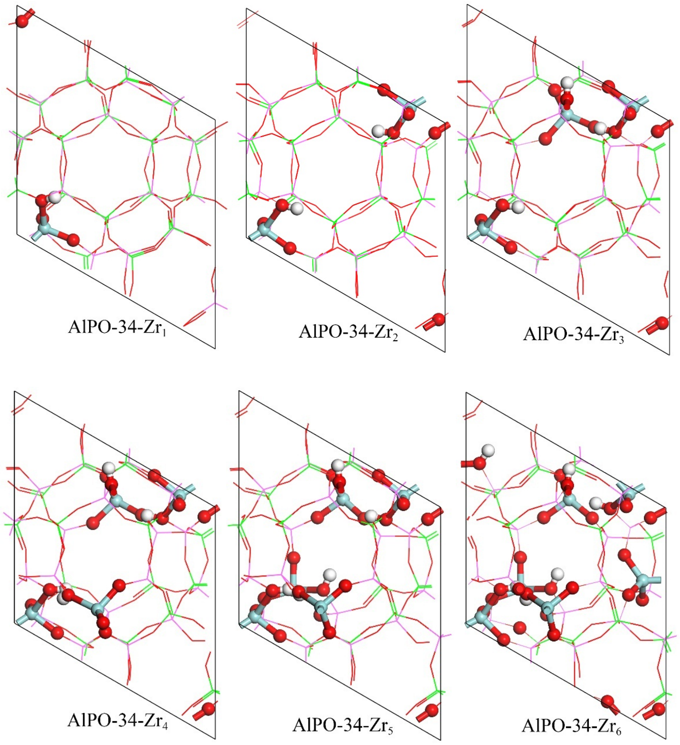

Figure S4. The most stable configurations of AlPO-34-$Zr_{1\text{-}6}$.

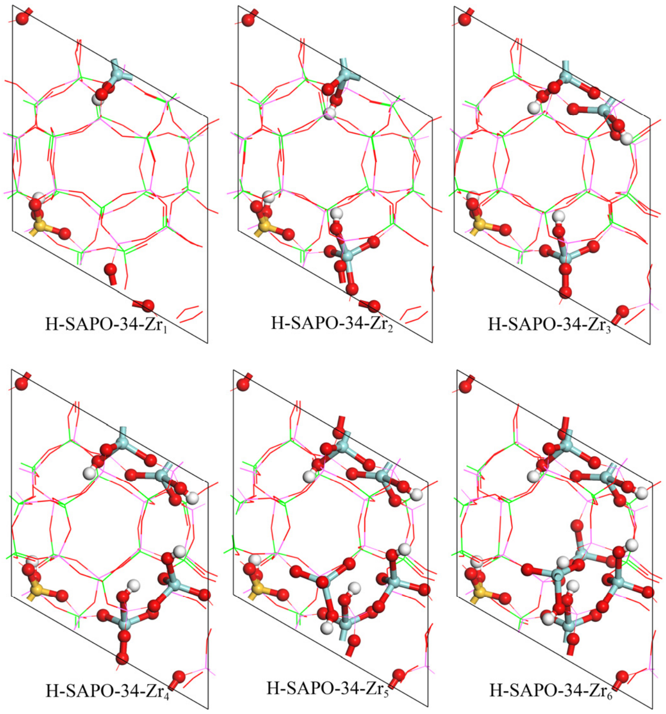

Figure S5. The most stable configurations of SAPO-34-Zr_{1-6}.

References

1. Deka, R.C.; Nasluzov, V.A.; Ivanova Shor, E.A.; Shor, A.M.; Vayssilov, G.N.; Rösch, N. Comparison of All Sites for Ti Substitution in Zeolite TS-1 by an Accurate Embedded-Cluster Method. *J. Phys. Chem. B* **2005**, *109*, 24304–24310.
2. Chatterjee, A.; Mizukami, F. Location and role of exchangeable cations in zeolite catalysis: A first principle study. *Chem. Phys. Lett.* **2004**, *385*, 20–24.
3. Dong, J.; Zhu, H.; Xiang, Y.; Wang, Y.; An, P.; Gong, Y.; Liang, Y.; Qiu, L.; Zheng, A.; Peng, X.; et al. Toward a unified identification of Ti location in the MFI framework of high-Ti-loaded TS-1: Combined EXAFS, XANES, and DFT Study. *J. Phys. Chem. C* **2016**, *120*, 20114–20124.
4. Xing, B.; Ma, J.; Li, R.; Jiao, H. Location, distribution and acidity of Al substitution in ZSM-5 with different Si/Al ratios—a periodic DFT computation. *Catal. Sci. Technol.* **2017**, *7*, 5694–5708.
5. Jones, A.J.; Iglesia, E. The Strength of Brønsted Acid Sites in Microporous Aluminosilicates. *ACS Catal.* **2015**, *5*, 5741–5755.
6. Gamba, A.; Tabacchi, G.; Fois, E. TS-1 from First Principles. *J. Phys. Chem. A* **2009**, *113*, 15006–15015.
7. Yang, G.; Wang, Y.; Zhou, D.; Liu, X.; Han, X.; Bao, X. Density functional theory calculations on various M/ZSM-5 zeolites: Interaction with probe molecule H2O and relative hydrothermal stability predicted by binding energies. *J. Mol. Catal. A: Chem.* **2005**, *237*, 36–44.
8. Montejo-Valencia, B.D.; Curet-Arana, M.C. DFT Study of the Lewis Acidities and Relative Hydrothermal Stabilities of BEC and BEA Zeolites Substituted with Ti, Sn, and Ge. *J. Phys. Chem. C* **2015**, *119*, 4148–4157.
